# Machine learning model for predicting hypotension following continuous renal replacement therapy initiation in end-stage kidney disease patients: a SHAP-interpretable approach

**DOI:** 10.3389/fmed.2026.1807513

**Published:** 2026-05-15

**Authors:** Shuang Qiu, Dongxia Qiu, Yongyuan Tao, Panpan Fu, Jiuxu Bai, Ning Cao

**Affiliations:** 1 State Key Laboratory of Frigid Zone Cardiovascular Disease, Cardiovascular Research Institute and Department of General Medicine, General Hospital of Northern Theater Command, Shenyang, China; 2 Department of Blood Purification, General Hospital of Northern Theater Command, Shenyang, China

**Keywords:** continuous renal replacement therapy, end-stage kidney disease, intradialytic hypotension, machine learning, prediction model

## Abstract

**Background:**

Early prediction of intradialytic hypotension (IDH) after starting continuous renal replacement therapy (CRRT) is critical for timely intervention. However, effective models for predicting the risk of IDH in patients with end-stage kidney disease (ESKD) undergoing CRRT are currently lacking. Therefore, the aim of this study was to develop a machine learning (ML)-based predictive model to facilitate the early identification of high-risk patients and to support clinical decision-making.

**Methods:**

Adult patients with ESKD who underwent CRRT were enrolled in this study and randomly divided into training (70%) and testing sets (30%). IDH was defined as a reduction in systolic blood pressure (SBP) ≥ 20 mmHg from baseline within 6 h after CRRT initiation; supplementary definitions were also applied, including a decrease in SBP ≥ 30 mmHg or in mean arterial pressure (MAP) ≥ 10 mmHg from baseline. Demographic characteristics, medication use, laboratory parameters, and treatment-related variables were collected. Multiple ML algorithms—including gradient boosting machine (GBM), extreme gradient boosting (XGBoost), decision tree (DT), support vector machine (SVM), random forest (RF), and logistic regression (LR)—were used to develop predictive models. Model performance was evaluated using the area under the receiver operating characteristic curve (AUC) and other performance metrics. Shapley additive explanations (SHAP) was applied to quantify the contribution of each feature to the model predictions.

**Results:**

Overall, 1,103 patients were included. Within the dataset used in this study, the SVM model consistently outperformed the other compared algorithms across all definitions of IDH, with an AUC of 0.805, indicating good calibration and clinical utility. The optimized simplified model retained stable predictive capacity (AUC = 0.809). SHAP analysis revealed that SBP was the most important feature for predicting IDH.

**Conclusion:**

The results of this study demonstrate the effectiveness of several ML algorithms in predicting the risk of IDH following the initiation of CRRT in patients with ESKD. The SVM model yielded the most favorable predictive performance in our comparative analysis. SBP was identified as a key predictor of IDH. The proposed model can assist in the clinical identification of high-risk patients and facilitate timely interventions.

## 1 Introduction

Chronic kidney disease (CKD) has become a significant global public health issue, with increasing morbidity and mortality rates and healthcare burdens. Patients may eventually progress to end-stage kidney disease (ESKD), requiring renal replacement therapy to sustain life. According to epidemiological data from the Global Burden of Disease (GBD) study and the International Society of Nephrology (ISN), the global prevalence of ESKD is approximately 10–12 million, and 3–4 million new cases are identified annually (1). Among patients requiring renal replacement therapy, approximately 70–80% (350–400 million) undergo maintenance haemodialysis (2). Haemodialysis therapies primarily include intermittent haemodialysis and continuous renal replacement therapy (CRRT) (3). Due to its ability to provide a more stable haemodynamic environment, CRRT has become a crucial treatment for critically ill patients, particularly those with haemodynamic instability (4). However, ESKD patients often present with complex conditions and multiple comorbidities, making them prone to experiencing new clinical events during treatment. Therefore, the early identification of and interventions for related complications are clinically important for improving patient outcomes and survival rates.

Intradialytic hypotension (IDH) is a common complication during dialysis treatment. In patients with ESKD undergoing routine intermittent haemodialysis, the incidence of IDH is approximately 11% (5). In contrast, among patients with acute kidney injury (AKI) receiving CRRT, the incidence can reach approximately 40% (6, 7). However, there is currently a lack of reports specifically on the incidence of IDH in the ESKD population undergoing CRRT. Despite the relatively mild haemodynamic impact of CRRT, these critically ill ESKD patients remain at high risk for IDH due to baseline circulatory instability. The occurrence of IDH is associated with increased all-cause mortality, cardiovascular mortality, and readmission risk (8, 9); it can lead to reduced blood perfusion in major organs, thereby increasing the risk of adverse events such as myocardial infarction, stroke, and arteriovenous fistula thrombosis (10, 11). Consequently, developing a reliable IDH risk prediction model for ESKD patients undergoing CRRT is important. Such a model would aid in the early identification of high-risk patients, facilitate risk stratification, guide individualized interventions, reduce the risk of organ perfusion deficits and related thrombotic events, ensure continuity of treatment, and ultimately improve clinical outcomes and quality of life for patients.

Despite the well-established harms of IDH, accurate prediction remains challenging due to the complex and heterogeneous nature of patients' conditions (12). In recent years, ML techniques have demonstrated significant advantages in handling complex clinical data and constructing predictive models (13). ML has been applied to predict IDH in patients with AKI undergoing CRRT (6) and in ESKD patients undergoing intermittent haemodialysis (14). However, there are essential differences in the mechanisms of IDH between ESKD and AKI patients, such as vascular compliance, diastolic dysfunction, volume load, underlying diseases, vascular permeability, and autonomic nervous regulation. Furthermore, CRRT and intermittent haemodialysis differ in treatment principles and haemodynamic effects. Therefore, extrapolating predictive models developed for AKI or intermittent haemodialysis populations to ESKD patients undergoing CRRT may have significant limitations in applicability. Although previous studies have explored IDH prediction in AKI and ESKD patients under different dialysis modes, no studies have specifically focused on predicting the risk of IDH during CRRT in ESKD patients. Given the significant advantages of ML in handling complex clinical data and constructing predictive models, the aim of this study was to develop and validate an ML model specifically for predicting the risk of IDH in ESKD patients receiving CRRT to subsequently enable early warning and risk stratification.

## 2 Methods

### 2.1 Data source and study population

**Figure 1:**
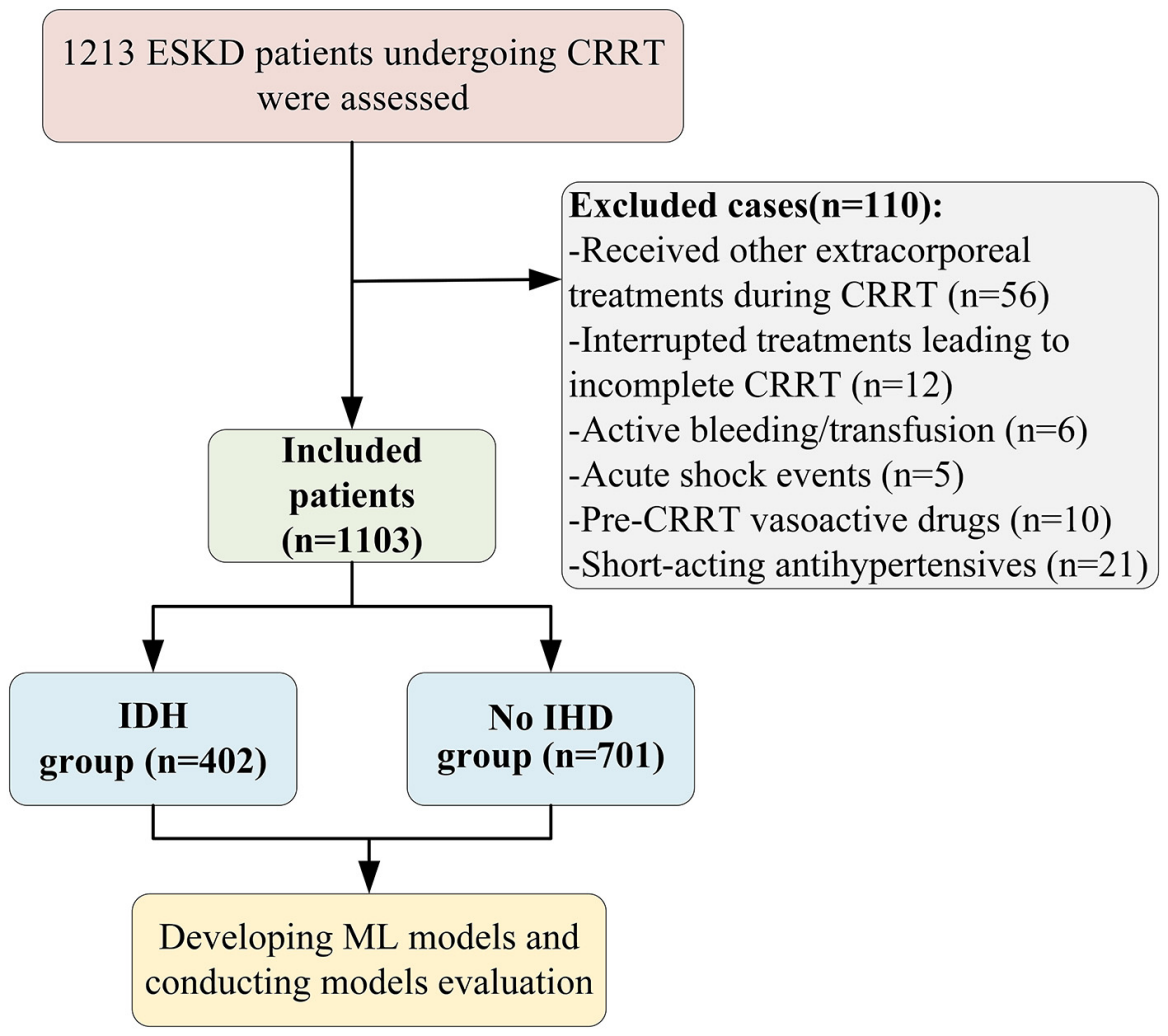
Flow chart of the study patients. ESKD, end-stage kidney disease; CRRT, continuous renal replacement therapy; IDH, intradialytic hypotension; ML, machine learning.

This retrospective cohort study included 1,213 ESKD patients who received CRRT at the Department of Blood Purification, General Hospital of the Northern Theater Command, between January 1, 2023, and September 30, 2024. Nephrologists assessed the need for CRRT in ESKD patients on the basis of various clinical factors, including haemodynamic instability, multiple organ dysfunction, hyperkalaemia, hypernatraemia, severe metabolic acidosis, cerebral oedema, or a hypercatabolic state (4). The inclusion criteria were as follows: (1) age ≥18 years, (2) estimated glomerular filtration rate (eGFR) <15 mL/min/1.73 m^2^ (calculated using the CKD-EPI equation) in accordance with the KDIGO guidelines (3), and (3) a duration of CRRT ≥ 6 h. The exclusion criteria were as follows: (1) other extracorporeal treatments during CRRT (e.g., plasmapheresis, haemoperfusion, or extracorporeal membrane oxygenation); (2) incomplete CRRT treatment because of death or other reasons; (3) active massive bleeding or the need for blood transfusion; (4) incident acute myocardial infarction, severe arrhythmias, pulmonary embolism, or severe allergic reactions leading to shock during treatment; (5) previous prescriptions of continuous infusions of vasopressors (such as norepinephrine, dopamine, dobutamine, or vasopressin) prior to the initiation of CRRT and the need to continue on these treatments to maintain baseline blood pressure after the initiation of CRRT; and (6) the use of short-acting antihypertensive medications (e.g., sodium nitroprusside, nicardipine, captopril, and nifedipine). A total of 1,103 patients were included in the final analysis (Figure 1). Three widely used and clinically meaningful definitions of IDH were employed in this study: (1) an absolute decrease in systolic blood pressure (SBP) ≥ 20 mmHg (15), (2) an absolute decrease in SBP ≥ 30 mmHg (16), and (3) a decrease in mean arterial pressure (MAP) ≥ 10 mmHg (15) from baseline. Given that some patients underwent multiple CRRT sessions during hospitalization, only the first CRRT session of each patient was included to avoid duplication and ensure data accuracy. This study followed the ethical principles outlined in the Declaration of Helsinki (2024 revised version) and was approved by the Ethics Committee of the General Hospital of the Northern Theater Command (Ethics Approval No: Y-2025-028). As a retrospective cohort study, the Strengthening the Reporting of Observational Studies in Epidemiology (STROBE) guidelines were followed to ensure transparency and scientific rigor in the design and reporting. Since this was a retrospective observational study, the requirement for obtaining written informed consent from each patient was waived.

### 2.2 Study variables and outcomes

In this study, general demographic data, medical history and medication information, vital signs, CRRT treatment prescriptions, and laboratory test results were collected prior to the initiation of CRRT to establish and evaluate the performance of different models. The demographic information included sex, age, and weight. CRRT treatment data included the type of blood dialysis filter (Gambro ST100, ST150, and M100); vascular access for dialysis, which included arteriovenous fistulas (punctured using a 16G needle, approximately equivalent to 1.65 mm in diameter), standard double-lumen internal jugular vein dialysis catheters (11.5 Fr, 13 cm), standard double-lumen femoral vein dialysis catheters (11.5 Fr, 20 cm), a right internal jugular vein chronic catheter (tunneled, 14.5 Fr, 19 cm), a left internal jugular vein chronic catheter (tunneled, 14.5 Fr, 14.5 Fr, 23 cm), and a femoral vein chronic catheter (tunneled, 14.5 Fr, 33 cm); treatment model (CVVH); blood flow rate (150–250 ml/min); replacement fluid (Yishuke, Shijiazhuang No. 4 Pharmaceutical, containing glucose, sodium chloride, magnesium chloride, and calcium chloride); ultrafiltration quantity; dose (20–25 mL/kg); anticoagulant use; potassium in replacement fluid; and sodium bicarbonate infusion rate during CRRT. Vital signs collected at the initiation of CRRT included heart rate, SBP, diastolic blood pressure (DBP), and MAP. Laboratory test results prior to the initiation of CRRT included white blood cell count; neutrophil ratio; lymphocyte ratio; red blood cell count; hemoglobin; haematocrit; platelet count; platelet distribution width; direct bilirubin; indirect bilirubin; aspartate aminotransferase; alanine aminotransferase; alkaline phosphatase; albumin; blood urea nitrogen; creatinine; uric acid; glucose; calcium; phosphate; potassium; sodium; chloride; carbon dioxide; cholesterol; triglycerides; high-density lipoprotein; low-density lipoprotein; prothrombin time; international normalized ratio; p-prothrombin time activity; activated partial thromboplastin time; fibrinogen; thrombin time; D-dimer; and intact parathyroid hormone. In addition, data on the ejection fraction, dialysis time, and comorbidities, such as hypertension, diabetes mellitus, coronary heart disease, arrhythmia, and stroke, and the use of antihypertensive medications (oral calcium channel blocker (CCB), oral angiotensin-converting enzyme inhibitor/angiotensin II receptor blocker (ACEI/ARB), and oral β-blockers) were also collected. The primary outcome of the study was the occurrence of IDH after the initiation of CRRT. IDH was defined according to three common clinical standards: (1) a decrease in SBP ≥ 20 mmHg from baseline within 6 h; (2) a decrease in SBP ≥ 30 mmHg from baseline within 6 h; and (3) a decrease in MAP ≥ 10 mmHg from baseline within 6 h. Data for this study were obtained from the standardized inpatient information system and certified laboratory database of the study hospital. Specifically, CRRT-related data were extracted from strictly maintained paper records and cross-verified by a dedicated medical team. Demographic characteristics, vital signs, laboratory test results, and medical history were obtained from the aforementioned electronic systems. To ensure the highest data quality, all data entry and integration were performed using a dual independent entry and verification system.

### 2.3 Statistical analysis

For continuous variables, the Shapiro–Wilk test was initially used to assess normality. If both groups followed a normal distribution, Levene's test was conducted to check for homogeneity of variance. On the basis of the results, either Student's *t-*test or Welch's *t*-test was applied. If the normality assumption was not met, the Mann–Whitney *U*-test was used. Continuous variables are described as the median (25th percentile−75th percentile). Categorical variables were analyzed using contingency tables; Fisher's exact test was applied when the expected frequency in any cell was <5; otherwise, the chi-square test was used. Categorical variables are summarized as frequencies and percentages. All the statistical tests were two-tailed, and the significance level was set at 0.05.

### 2.4 Data preprocessing

As the missing data were missing at random, multiple imputation by chained equations was used to impute the missing values (17). This method generates multiple imputed datasets through an iterative conditional regression model and combines the results in subsequent analyses using Rubin's rules. This process aims to maximize the use of available data and enhance the robustness of statistical inferences. To ensure rigorous analysis, the imputation process for the training and testing sets was conducted independently, avoiding the risk of data leakage and ensuring the reliability of the results. The consistency of the imputed data distributions with the original observed values was assessed using independent samples *t*-tests (for numerical variables) or chi-square tests (for categorical variables). On this basis, variables with small variance (<0.01) were excluded (18). To reduce the instability caused by multicollinearity, we removed features whose absolute correlation coefficient was greater than 0.9 with any other variable (19) and those whose variance inflation factor (VIF) was greater than 10 (20).

### 2.5 Model development

After data preprocessing, the dataset was randomly split into a training set and a testing set at a 7:3 ratio. Six ML algorithms were employed to construct the models, namely, gradient boosting machine (GBM), extreme gradient boosting (XGBoost), decision tree (DT), support vector machine (SVM), random forest (RF), and logistic regression (LR). Models were developed with a focus on both predictive performance and interpretability, with a systematic evaluation of their applicability in clinical prediction. For each ML model, hyperparameter tuning was performed exclusively on the training set using a 5-fold cross-validation combined with a grid search strategy. Hyperparameter tuning was performed only on the training set to prevent data leakage. Specifically, the training set was divided into five mutually exclusive folds. In each iteration, 4-folds were used for training and the remaining fold was used for validation. The grid search traversed a predefined set of hyperparameter combinations, and the combination yielding the highest mean cross-validated AUC was selected as the optimal hyperparameter set, details are provided in Supplementary Figure 1. This entire procedure was conducted within the training set only, without any access to the testing set or the temporal validation set, thereby preventing data leakage. Details of the hyperparameter tuning for each algorithm are provided in Supplementary Table 1. To mitigate the potential impact of class imbalance, the class_weight = “balanced” parameter was applied during model training. This approach adjusts the penalty weights for misclassification, assigning higher weights to the minority class (IDH-positive patients) inversely proportional to class frequencies, thereby reducing the bias that could otherwise favor the majority class. Given that this study is based on a single-center retrospective design and lacks external validation, we adopted a temporal validation strategy to provide a more robust assessment of model generalizability. The dataset was chronologically split into training and testing sets according to patient admission date: data from January 1, 2023 to February 29, 2024 were used as the training set (*n* = 779), and data from March 1, 2024 to September 30, 2024 were used as a temporally independent testing set (*n* = 324). The six ML algorithms described above were, respectively employed to develop predictive models, and relevant performance metrics were calculated for evaluation.

To further simplify the models and enhance computational efficiency, least absolute shrinkage and selection operator (LASSO) regression was used for feature selection (21). To avoid data leakage, all feature selection and regularization procedures were conducted solely within the training set, with no access to the test set at any stage. Five fold cross-validation was performed on the training set, and the regularization parameter λ was automatically searched within the logarithmic scale range (10^−4^ to 10^4^). The λ that minimized the mean squared error (λmin) was selected for feature selection and variable importance scoring.

### 2.6 Model evaluation

To comprehensively assess model performance, the area under the receiver operating characteristic curve (AUC), sensitivity, specificity, positive predictive value (PPV), negative predictive value (NPV), and F1 score were calculated. To assess the statistical differences in predictive performance among different ML models, the DeLong test was employed to perform pairwise comparisons of the AUC between each model and the model with the highest AUC. The significance level was set at *P* < 0.05. Considering the inherent class imbalance across different IDH definitions (incidence: 19.4%−46.5%), we further calculated AUPRC as a complementary evaluation indicator. Combined with class weighting strategy, AUPRC was applied to objectively quantify and reduce the confounding effect of imbalanced sample distribution on model performance. Decision curve analysis (DCA) was used to evaluate the net benefit of the model at different thresholds, thus measuring its practical applicability. The calibration performance of the model was visualized using calibration curves, and the Brier score (which ranged from 0 to 1) was computed, with lower values indicating better calibration performance. To further quantify the calibration of the model, the calibration intercept and calibration slope were used. Shapley additive explanation (SHAP) values (22, 23) were used to interpret the “black box” nature of the models, quantifying and visualizing the relative contribution of each feature to the model predictions. To evaluate the stability of feature importance, a bootstrap resampling procedure was performed. Briefly, 500 bootstrap samples were generated by random resampling with replacement from the original dataset. The model was retrained on each bootstrap sample, and feature importance was recalculated independently for each iteration. The mean value and standard deviation of feature importance across all bootstrap runs were used to quantify the relative stability of each feature.

## 3 Results

### 3.1 Baseline characteristics

A total of 1,103 patients were included in this study, with the incidence of IDH varying according to the definition used. When IDH was defined as a decrease in MAP ≥10 mmHg within 6 h, a decrease in SBP ≥20 mmHg within 6 h, or a decrease in SBP ≥30 mmHg within 6 h, the incidence of IDH was 46.5% (513/1,103), 36.4% (401/1,103) and 19.4% (214/1,103), respectively. The median age of the patients was 61.0 (53.0–70.0) years, and 773 (70.1%) were male. The baseline SBP of the patients was 148.0 (129.0–166.0) mmHg, and the DBP was 78.8 (63.4–94.2) mmHg. All patients were randomly divided into a training set (*n* = 772) and a testing set (*n* = 331), with no statistically significant differences observed between the two groups for any variable characteristics (see Table 1).

**Table 1:** Baseline characteristics of the included ESKD patients.

Characteristic	Test (*n* = 331)	Train (*n* = 772)	Total (*n* = 1,103)	*P* value
Male, *n* (%)	237 (71.6%)	536 (69.4%)	773 (70.1%)	0.516
Age (years)	62.0 (54.0–71.0)	61.0 (52.0–70.0)	61.0 (53.0–70.0)	0.236
SBP (mmHg)	148.0 (128.0–165.0)	148.0 (129.0–166.0)	148.0 (128.0–165.0)	0.616
DBP (mmHg)	77.9 (62.6–93.2)	79.1 (63.7–94.5)	78.8 (63.3–94.2)	0.204
Weight (kg)	65.0 (56.0–75.0)	65.0 (55.5–75.0)	65.0 (56.0–75.0)	0.727
Vascular access, *n* (%)				0.559
Dialysis arteriovenous fistulas	193 (58.3%)	423 (54.8%)	616 (55.8%)	
Internal jugular vein dialysis catheter	92 (27.8%)	233 (30.2%)	325 (29.5%)	
Femoral venous dialysis catheter	46 (13.9%)	116 (15.0%)	162 (14.7%)	
Blood dialysis filter, *n* (%)				0.081
ST100	264 (79.8%)	579 (75%)	843 (76.4%)	
ST150	24 (7.3%)	98 (12.7%)	122 (11.1%)	
M100	43 (13.0%)	95 (12.3%)	138 (12.5%)	
Heart rate (beats/minute)	80.0 (70.0–91.0)	80.0 (70.0–91.2)	80.0 (70.0–91.0)	0.817
Potassium in replacement fluid (mmol/L)	3.4 (2.7–3.4)	3.4 (2.0–3.4)	3.4 (2.3–3.4)	0.369
Sodium bicarbonate infusion rate during CRRT (ml/h)	150.0 (135.0–150.0)	150.0 (135.0–150.0)	150.0 (135.0–150.0)	0.173
Treatment model (CVVH), n (%)	141 (42.6%)	355 (46%)	496 (45%)	0.567
Blood flow rate (ml/min)	180 (180.0–180.0)	180 (180.0–180.0)	180 (180.0–180.0)	0.387
Predilution replacement fluid (ml/h)	1,800.0 (1,800.0–1,800.0)	1,800.0 (1,800.0–1,800.0)	1,800.0 (1,800.0–1,800.0)	0.835
Postdilution replacement fluid (ml/h)	600.0 (400.0–600.0)	600.0 (300.0–600.0)	600.0 (400.0–600.0)	0.346
Ultrafiltration quantity (ml/h)	375.0 (265.9–490.0)	416.7 (311.8–500.0)	416.7 (300–500)	0.108
White blood cell count ( × 10^9^/L)	6.5 (5.3–9.1)	6.7 (5.2–8.9)	6.6 (5.3–9.0)	0.945
Neutrophil ratio (%)	76.8 (69.5–83.4)	77.2 (69.8–83.5)	77.0 (69.7–83.5)	0.488
Lymphocyte ratio (%)	13.1 (8.2–18.6)	12.9 (8.1–18.4)	13.0 (8.1–18.5)	0.46
Red blood cell count ( × 10^12^/L)	3.2 (2.7–3.9)	3.2 (2.6–3.8)	3.2 (2.6–3.8)	0.254
Platelet count ( × 10^9^/L)	168.0 (127.0–226.5)	173.0 (125.0–227.0)	171.0 (126.0–227.0)	0.801
Platelet distribution width	16.2 (15.9–16.5)	16.2 (15.9–16.5)	16.2 (15.9–16.5)	0.812
Direct bilirubin (umol/L)	1.9 (1.3–3.0)	2.0 (1.3–3.2)	2.0 (1.3–3.1)	0.148
Indirect bilirubin (umol/L)	3.4 (2.4–4.8)	3.4 (2.3–4.9)	3.4 (2.3–4.9)	0.835
Aspartate aminotransferase (U/L)	15.5 (11.1–22.3)	16.6 (11.3–24.9)	16.3 (11.2–24.2)	0.112
Alanine aminotransferase (U/L)	12.3 (8.5–19.5)	12.5 (8.2–21.3)	12.4 (8.3–21.3)	0.523
Alkaline phosphatase (U/L)	82.7 (64.4–104.0)	84.9 (66.5–113.1)	84.0 (66.0–109.7)	0.091
Albumin (g/L)	35.7 (30.7–39.1)	34.7 (30.2–38.7)	35.0 (30.3–38.9)	0.079
Blood urea nitrogen (mmol/L)	22.4 (17.3–29.8)	23.3 (17.0–29.9)	23.0 (17.0–29.9)	0.863
Creatinine (umol/L)	781.2 (566.6–993.2)	751.6 (565.8–1,002.2)	762.1 (566.0–1001.4)	0.661
Uric acid (umol/L)	407.8 (313.5–499.5)	407.4 (334.8–490.0)	407.8 (325.0–493.0)	0.960
Glucose (mmol/L)	7.0 (5.4–9.1)	7.0 (5.4–9.3)	7.0 (5.4–9.2)	0.684
Calcium (mmol/L)	2.1 (2.0–2.3)	2.1 (2.0–2.3)	2.1 (2.0–2.3)	0.714
Phosphate (mmol/L)	1.8 (1.4–2.3)	1.8 (1.4–2.3)	1.8 (1.4–2.3)	0.460
Potassium (mmol/L)	4.6 (4.1–5.2)	4.6 (4.1–5.3)	4.6 (4.1–5.2)	0.854
Sodium (mmol/L)	138.0 (135.4–140.8)	137.9 (135.0–140.1)	137.9 (135.1–140.4)	0.077
Chloride (mmol/L)	100.0 (96.8–103.4)	99.6 (96.4–102.5)	99.6 (96.4–103.1)	0.159
Carbon dioxide (mmol/L)	22.6 (18.6–25.6)	22.1 (18.7–25.5)	22.3 (18.7–25.5)	0.864
Cholesterol (mmol/L)	3.8 (3.1–4.5)	3.8 (3.1–4.5)	3.8 (3.1–4.5)	0.713
Triglycerides (mmol/L)	1.6 (1.1–2.1)	1.6 (1.1–2.1)	1.6 (1.1–2.1)	0.932
High–density lipoprotein (mmol/L)	1.0 (0.8–1.2)	0.9 (0.7–1.2)	0.9 (0.7–1.2)	0.059
Low–density lipoprotein (mmol/L)	2.0 (1.5–2.4)	1.9 (1.5–2.5)	1.9 (1.5–2.4)	0.866
Prothrombin time (s)	12.6 (11.8–13.6)	12.7 (11.8–13.8)	12.6 (11.8–13.7)	0.715
p–Prothrombin time activity (%)	92.1 (76.9–105.0)	92.1 (77.9–107.0)	92.1 (77.4–106.4)	0.745
Activated partial thromboplastin time (s)	29.1 (26.4–34.6)	29.4 (26.4–34.2)	29.2 (26.4–34.3)	0.926
Fibrinogen (g/L)	4.0 (3.2–5.1)	4.2 (3.2–5.3)	4.1 (3.2–5.2)	0.459
Thrombin time (s)	17.6 (16.9–18.5)	17.6 (16.8–18.5)	17.6 (16.8–18.5)	0.798
D–dimer (mg/L)	1.3 (0.6–2.9)	1.4 (0.7–3.2)	1.4 (0.6–3.2)	0.193
Intact parathyroid hormone (pg/mL)	410.4 (189.0–631.0)	378.0 (176.8–588.0)	382.0 (178.0–610.5)	0.38
Ejection fraction	0.6 (0.5–0.6)	0.6 (0.5–0.6)	0.6 (0.5–0.6)	0.800
Dialysis time (month)	24.0 (0–72.0)	24.0 (0–72.0)	24.0 (0–72.0)	0.81
Anticoagulant use, *n* (%)	253 (76.4%)	566 (73.3%)	819 (74.3%)	0.312
Hypertension, *n* (%)	306 (92.4%)	713 (92.4%)	1,019 (92.4%)	0.998
Diabetes mellitus, *n* (%)	161 (48.6)	402 (52.1%)	563 (51.0%)	0.327
Coronary heart disease, *n* (%)	128 (38.7%)	297 (38.5%)	425 (38.5%)	0.999
Arrhythmia, *n* (%)	42 (12.7%)	83 (10.8%)	125 (11.3%)	0.408
Stroke, *n* (%)	71 (21.5%)	160 (20.7%)	231 (20.9%)	0.849
Oral CCB, *n* (%)	139 (42.0%)	351 (45.5%)	490 (44.4%)	0.318
Oral ACEI/ARB, *n* (%)	155 (46.8%)	381 (49.4%)	536 (48.6%)	0.482
Oral β-blocker, *n* (%)	153 (46.2%)	370 (47.9%)	523 (47.4%)	0.650

ESKD, end-stage kidney disease; SBP, systolic blood pressure; DBP, diastolic blood pressure; CRRT, continuous renal replacement therapy; CVVH, continuous venovenous haemofiltration; CCB, calcium channel blocker; ACEI/ARB, angiotensin-converting enzyme inhibitor/angiotensin II receptor blocker.

### 3.2 Variable filtering and preprocessing results

The proportions of missing values for each variable are presented in Supplementary Table 2. After multiple imputation, the distributions of the imputed variables were consistent with those of the original data. A low-variance filter (threshold = 0.01) was subsequently applied, which removed haematocrit (variance = 0.005) from consideration. During the collinearity analysis, strong correlations were identified between MAP and DBP, hemoglobin and the red blood cell count, and the international normalized ratio and prothrombin time, with correlation coefficients of 0.907, 0.908, and 0.927, respectively, all exceeding the predefined threshold of 0.9. On the basis of clinical interpretability, common use, pathophysiological relevance, and a reduction in information redundancy, MAP (as it can be derived from DBP and SBP), hemoglobin (as it is highly dependent on the red blood cell count and is more commonly regarded as a diagnostic indicator of anemia rather than a causal variable), and the international normalized ratio (as a standardized derivative of the prothrombin time) were excluded from further analysis. Finally, VIF analysis was performed on the remaining variables. All the retained variables exhibited VIF values less than 10, indicating the absence of significant multicollinearity (see Supplementary Table 3 for details).

### 3.3 Model performance comparisons

**Figure 2:**
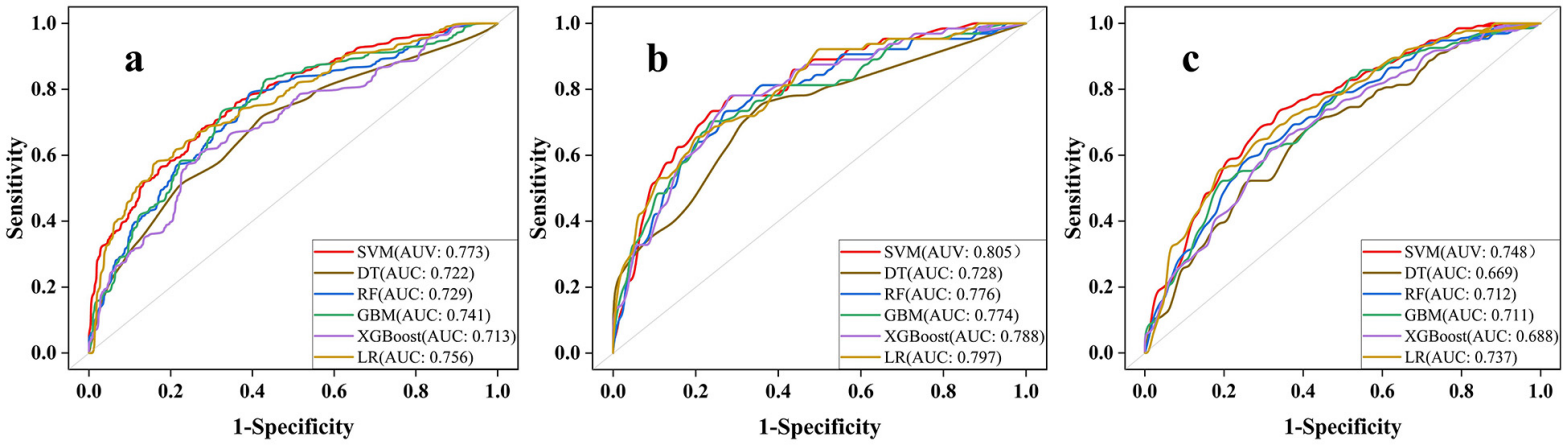
**(a)** ROC curves generated by the six models with the outcome defined as a reduction in SBP ≥ 20 mmHg from baseline. **(b)** ROC curves generated by the six models, with the outcome defined as a reduction in SBP ≥ 30 mmHg from baseline. **(c)** ROC curves generated by the six models with the outcome defined as a reduction in MAP ≥ 10 mmHg from baseline. SVM, support vector machine; XGBoost, extreme gradient boosting; GBM, gradient boosting machine; RF, random forest; DT, decision tree; LR, logistic regression; ROC, receiver operating characteristic; AUC, the area under the receiver operating characteristic curve; SBP, systolic blood pressure; MAP, mean arterial pressure.

In this study, six ML models were developed to predict the risk of IDH in patients with ESKD after the initiation of CRRT, and their predictive performance was comprehensively evaluated. When IDH was defined as a reduction in SBP ≥ 20 mmHg from baseline within 6 h, the SVM model demonstrated the best performance in this comparison, with an AUC of 0.773 and an AUPRC of 0.724, followed by the LR model, with an AUC of 0.756. When a more stringent definition of IDH (an SBP decrease ≥ 30 mmHg within 6 h) was used, the SVM and LR models continued to show superior predictive ability, with AUCs of 0.805 and 0.797, respectively. When IDH was defined as a MAP reduction ≥10 mmHg within 6 h, the SVM model again outperformed the other models, with an AUC of 0.749, compared with 0.737 for the LR model and 0.712 for the RF model. Figure 2 presents the receiver operating characteristic (ROC) curves for all models, whereas their precision-recall curves are shown in Supplementary Figure 2. Detailed performance metrics are summarized in Tables 2–4.

**Table 2:** Model performance metrics (IDH defined as a reduction in SBP ≥ 20 mmHg from baseline).

Model	AUC (95% CI)	Sensitivity (95% CI)	Specificity (95% CI)	PPV (95% CI)	NPV (95% CI)	F1 score (95% CI)	Brier's score (95% CI)	Calibration intercept (95% CI)	Calibration slope (95% CI)	AUPRC (95% CI)
SVM	0.773 (0.718–0.821)	0.822 (0.736–0.908)	0.858 (0.811–0.903)	0.856 (0.762–0.951)	0.752 (0.670–0.805)	0.838 (0.760–0.917)	0.178 (0.161–0.198)	−0.036 (−0.092–0.020)	1.037 (0.978–1.096)	0.724 (0.662–0.786)
XGBoost	0.713 (0.650–0.771)	0.760 (0.655–0.855)	0.849 (0.802–0.897)	0.813 (0.709–0.917)	0.745 (0.691–0.799)	0.785 (0.697–0.873)	0.188 (0.176–0.214)	−0.035 (−0.131–0.061)	1.035 (0.934–1.136)	0.667 (0.598–0.736)
GBM	0.741 (0.686–0.797)	0.734 (0.643–0.826)	0.858 (0.811–0.902)	0.823 (0.713–0.933)	0.748 (0.693–0.804)	0.776 (0.687–0.865)	0.188 (0.168–0.210)	−0.018 (−0.106–0.070)	1.018 (0.923–1.114)	0.720 (0.660–0.780)
RF	0.729 (0.669–0.789)	0.763 (0.676–0.851)	0.821 (0.765–0.877)	0.812 (0.697–0.927)	0.727 (0.674–0.780)	0.787 (0.699–0.876)	0.192 (0.172–0.212)	−0.048 (−0.126–0.070)	1.065 (0.965–1.165)	0.706 (0.657–0.755)
DT	0.722 (0.662–0.780)	0.798 (0.701–0.891)	0.862 (0.817–0.906)	0.800 (0.690–0.911)	0.771 (0.721–0.822)	0.799 (0.711–0.888)	0.197 (0.182–0.211)	0.007 (−0.089–0.103)	0.993 (0.808–1.179)	0.711 (0.650–0.772)
LR	0.756 (0.696–0.809)	0.804 (0.715–0.893)	0.867 (0.818–0.908)	0.863 (0.763–0.964)	0.776 (0.724–0.829)	0.833 (0.754–0.912)	0.183 (0.164–0.204)	0.015 (−0.065–0.094)	0.960 (0.873–1.047)	0.716 (0.665–0.767)

IDH, intradialytic hypotension; SBP, systolic blood pressure; SVM, support vector machine; XGBoost, extreme gradient boosting; GBM, gradient boosting machine; RF, random forest; DT, decision tree; LR, logistic regression; AUC, area under the curve; PPV, positive predictive value; NPV, negative predictive value; AUPRC, area under the precision-recall curve.

**Table 3:** Model performance metrics (IDH defined as a reduction in SBP ≥ 30 mmHg from baseline).

Model	AUC (95% CI)	Sensitivity (95% CI)	Specificity (95% CI)	PPV (95% CI)	NPV (95% CI)	F1 score (95%CI)	Brier's score (95% CI)	Calibration intercept (95% CI)	Calibration slope (95% CI)	AUPRC (95% CI)
**SVM**	0.805 (0.740–0.862)	0.878 (0.822–0.934)	0.896 (0.851–0.941)	0.833 (0.787–0.879)	0.838 (0.773–0.903)	0.854 (0.805–0.903)	0.127 (0.105–0.149)	−0.013 (−0.075–0.049)	1.029 (0.960–1.098)	0.766 (0.714–0.818)
**XG Boost**	0.788 (0.718–0.846)	0.741 (0.653–0.829)	0.878 (0.809–0.947)	0.600 (0.537–0.663)	0.826 (0.767–0.885)	0.663 (0.620–0.706)	0.128 (0.107–0.149)	−0.035 (−0.127–0.057)	1.042 (0.929–1.155)	0.707 (0.646–0.768)
**GBM**	0.774 (0.704–0.838)	0.719 (0.650–0.788)	0.866 (0.803–0.929)	0.609 (0.573–0.645)	0.838 (0.792–0.884)	0.660 (0.611–0.709)	0.156 (0.104–0.153)	0.028 (−0.048–0.104)	0.929 (0.825–1.033)	0.711 (0.652–0.770)
**RF**	0.776 (0.712–0.835)	0.778 (0.770–0.856)	0.816 (0.767–0.865)	0.803 (0.717–0.889)	0.818 (0.759–0.877)	0.790 (0.721–0.859)	0.131 (0.110–0.153)	0.008 (−0.069–0.085)	0.979 (0.888–1.071)	0.734 (0.665–0.803)
**DT**	0.728 (0.656–0.799)	0.725 (0.629–0.822)	0.778 (0.682–0.874)	0.727 (0.658–0.797)	0.845 (0.796–0.894)	0.726 (0.632–0.820)	0.129 (0.106–0.153)	−0.009 (−0.095–0.076)	1.008 (0.888–1.128)	0.732 (0.681–0.783)
**LR**	0.797 (0.735–0.855)	0.825 (0.758–0.892)	0.885 (0.829–0.941)	0.767 (0.673–0.861)	0.824 (0.759–0.889)	0.795 (0.746–0.844)	0.127 (0.107–0.151)	0.010 (−0.062–0.082)	0.965 (0.876–1.054)	0.751 (0.705–0.797)

IDH, intradialytic hypotension; SBP, systolic blood pressure; SVM, support vector machine; XGBoost, extreme gradient boosting; GBM, gradient boosting machine; RF, random forest; DT, decision tree; LR, logistic regression; AUC, area under the curve; PPV, positive predictive value; NPV, negative predictive value.

**Table 4:** Model performance metrics (IDH defined as a reduction in MAP ≥ 10 mmHg from baseline).

Model	AUC (95% CI)	Sensitivity (95% CI)	Specificity (95% CI)	PPV (95% CI)	NPV (95% CI)	F1 score (95% CI)	Brier's score (95% CI)	Calibration intercept (95% CI)	Calibration slope (95% CI)	AUPRC (95% CI)
**SVM**	0.749 (0.691–0.803)	0.731 (0.652–0.809)	0.865 (0.819–0.911)	0.898 (0.845–0.951)	0.784 (0.738–0.830)	0.806 (0.760–0.852)	0.206 (0.187–0.224)	−0.016 (−0.053–0.021)	1.024 (0.976–1.071)	0.711 (0.646–0.776)
**XG Boost**	0.688 (0.624–0.748)	0.619 (0.536–0.708)	0.880 (0.816–0.944)	0.868 (0.830–0.906)	0.724 (0.655–0.793)	0.722 (0.665–0.779)	0.222 (0.206–0.239)	0.017 (−0.039–0.072)	0.962 (0.891–1.033)	0.643 (0.589–0.697)
**GBM**	0.711 (0.656–0.768)	0.642 (0.559–0.723)	0.814 (0.752–0.876)	0.831 (0.774–0.888)	0.716 (0.655–0.777)	0.724 (0.661–0.787)	0.216 (0.198–0.234)	0.022 (−0.060–0.105)	0.923 (0.805–1.040)	0.686 (0.630–0.742)
**RF**	0.712 (0.653–0.766)	0.649 (0.569–0.727)	0.855 (0.786–0.924)	0.861 (0.818–0.904)	0.733 (0.659–0.800)	0.740 (0.690–0.790)	0.218 (0.198–0.234)	−0.004 (−0.058–0.050)	0.985 (0.916–1.054)	0.677 (0.631–0.723)
**DT**	0.669 (0.610–0.726)	0.630 (0.584–0.676)	0.875 (0.833–0.917)	0.826 (0.740–0.912)	0.679 (0.603–0.755)	0.715 (0.651–0.779)	0.229 (0.217–0.241)	0.058 (−0.041–0.157)	0.902 (0.768–1.036)	0.652 (0.613–0.691)
**LR**	0.737 (0.684–0.790)	0.709 (0.627–0.785)	0.845 (0.784–0.906)	0.876 (0.809–0.943)	0.765 (0.715–0.815)	0.784 (0.720–0.848)	0.211 (0.193–0.229)	−0.024 (−0.080–0.032)	1.034 (0.957–1.110)	0.706 (0.645–0.767)

IDH, intradialytic hypotension; MAP, mean arterial pressure; SVM, support vector machine; XGBoost, extreme gradient boosting; GBM, gradient boosting machine; RF, random forest; DT, decision tree; LR, logistic regression; AUC, area under the curve; PPV, positive predictive value; NPV, negative predictive value; AUPRC, area under the precision-recall curve.

Pairwise comparisons of AUC values using the DeLong test further revealed that the SVM model significantly outperformed the other five ML models under all three IDH definitions, with all differences being statistically significant. These findings indicate that, within the context of our temporal validation framework, the SVM model provided more robust and stable predictive performance compared to the other five algorithms, details are shown in Supplementary Table 4.

As shown in Supplementary Table 5, in the validation set defined by admission time, the SVM model achieved an AUC of 0.778 (95% CI: 0.724–0.830) for predicting the primary outcome (a reduction in SBP of ≥ 20 mmHg), which was comparable to the AUC of 0.773 observed in the original internal validation. This finding indicates that the model exhibits good generalizability in the temporal dimension, without significant performance degradation.

**Figure 3:**
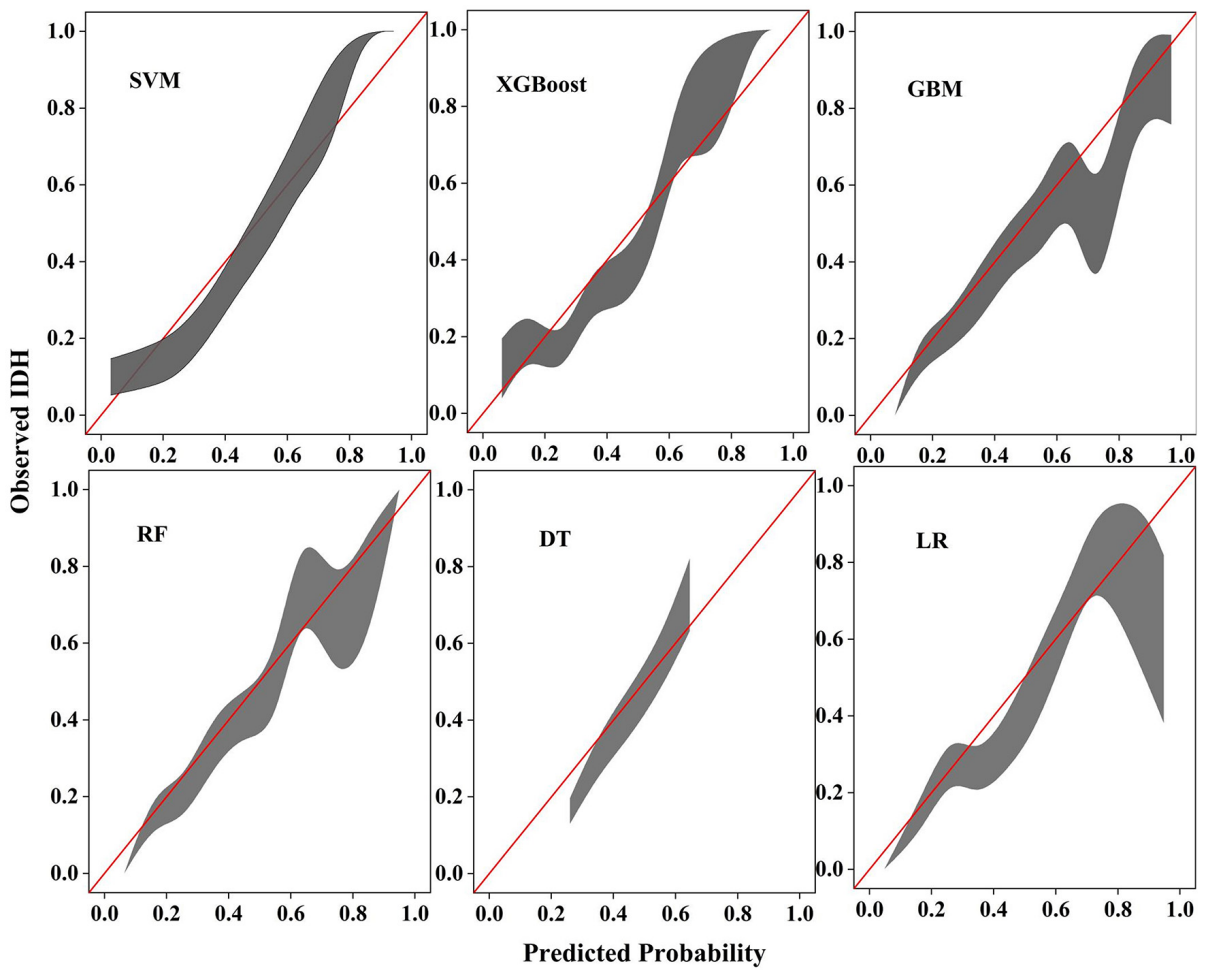
Calibration belts produced by the SVM, XGBoost, GBM, RF, DT, and LR models, with the outcome defined as a reduction in SBP ≥20 mmHg from baseline. The horizontal axis represents the model-predicted probability, and the vertical axis represents the observed event rate. The red diagonal line indicates the ideal calibration line (representing perfect agreement between predicted probability and actual observed outcome). The gray shaded area denotes the 95% confidence interval of each model's calibration curve, calculated based on the standard error of the actual outcome within each bin, reflecting the uncertainty of the model's calibration performance. SVM, support vector machine; XGBoost, extreme gradient boosting; RF, random forest; DT, decision tree; LR, logistic regression; GBM, gradient boosting machine.

**Figure 4:**
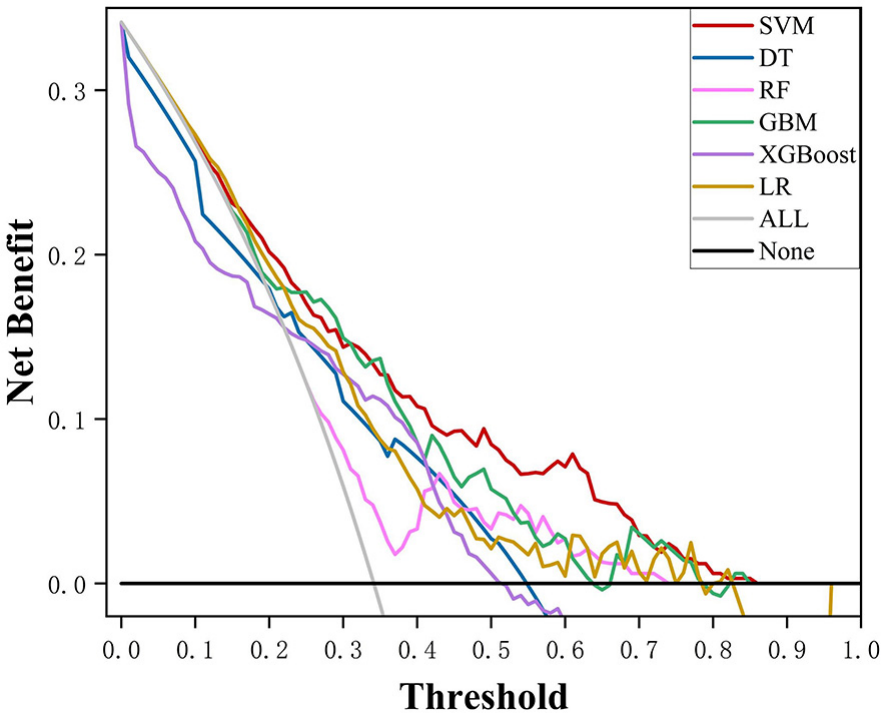
DCA results for the six models with the outcome defined as a reduction in SBP ≥20 mmHg from baseline. SVM, support vector machine; XGBoost, extreme gradient boosting; GBM, gradient boosting machine; RF, random forest; DT, decision tree; LR, logistic regression; DCA, decision curve analysis; SBP, systolic blood pressure.

To evaluate the calibration performance of the models, calibration curves were constructed, and Brier's scores were calculated. As shown in Figure 3, when IDH was defined as a reduction in SBP ≥ 20 mmHg from baseline within 6 h, the calibration curve of the SVM model showed good agreement with the ideal calibration line (diagonal line), indicating a high level of consistency between the predicted probabilities and the observed event rates. Under the other outcome definitions, the SVM model continued to demonstrate favorable calibration performance, as illustrated in Supplementary Figures 3 and 4. In addition, the Brier scores of the SVM model were 0.178, 0.127, and 0.206, respectively, which were all lower than those of the other comparative models, further supporting the accuracy of its predictions. Overall, the SVM model exhibited the best calibration performance in this study. DCA, as shown in Figure 4, demonstrated that the SVM model achieved greater net benefits across most threshold probability ranges, suggesting that the application of the SVM model in clinical practice may facilitate the effective identification of high-risk individuals.

### 3.4 Simplified model performance

**Figure 5:**
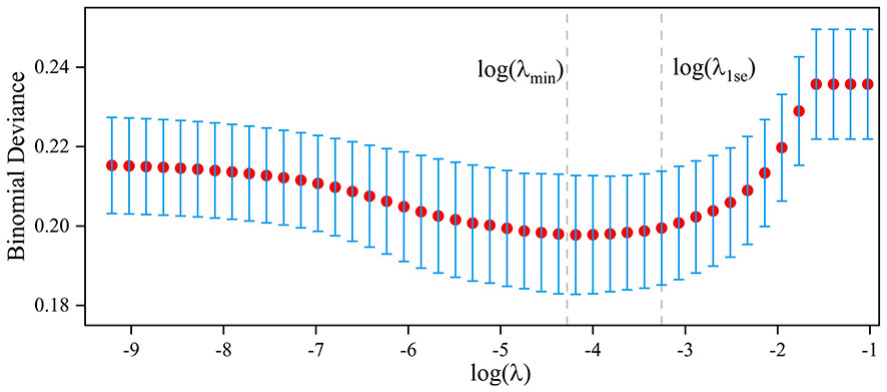
LASSO regularization path graph with the outcome defined as a reduction in SBP ≥20 mmHg from baseline. The log(λ1se) criterion was chosen to determine the variables used to construct models that were concise and had good predictive performance. LASSO, least absolute shrinkage and selection operator.

To simplify the model structure and improve computational efficiency, LASSO regression was further applied for variable refinement. When IDH was defined as a reduction in SBP ≥20 mmHg from baseline within 6 h, a total of 16 key variables were identified, including SBP, oral ACEI/ARB, red blood cell count, serum albumin concentration, oral calcium channel blocker use, thrombin time, glucose concentration, triglyceride concentration, prothrombin time, potassium concentration, platelet distribution width, phosphorus concentration, calcium concentration, alkaline phosphatase concentration, and platelet count, as shown in Figure 5. In this dataset, the SVM model achieved the best performance, with an AUC of 0.762, followed by the LR model (AUC = 0.756) and the GBM model (AUC = 0.738), as presented in Supplementary Figure 5. When the outcome was defined as a decrease in SBP ≥ 30 mmHg within 6 h, the selected variables included SBP, potassium in replacement fluid, calcium concentration, glucose concentration, prothrombin time, thrombin time, dialysis time, red blood cell count, blood flow rate, white blood cell count, and platelet distribution width. For the outcome defined as a MAP reduction ≥10 mmHg from baseline within 6 h, LASSO identified 15 important variables, including SBP, DBP, prothrombin time, creatinine concentration, calcium concentration, dialysis time, low-density lipoprotein concentration, red blood cell count, triglyceride concentration, heart rate, fibrinogen concentration, ultrafiltration quantity, thrombin time, oral ACEI/ARB use, and uric acid concentration. The performance of each model under different outcome definitions is summarized in Supplementary Tables 7–9.

### 3.5 Model explanations

**Figure 6:**
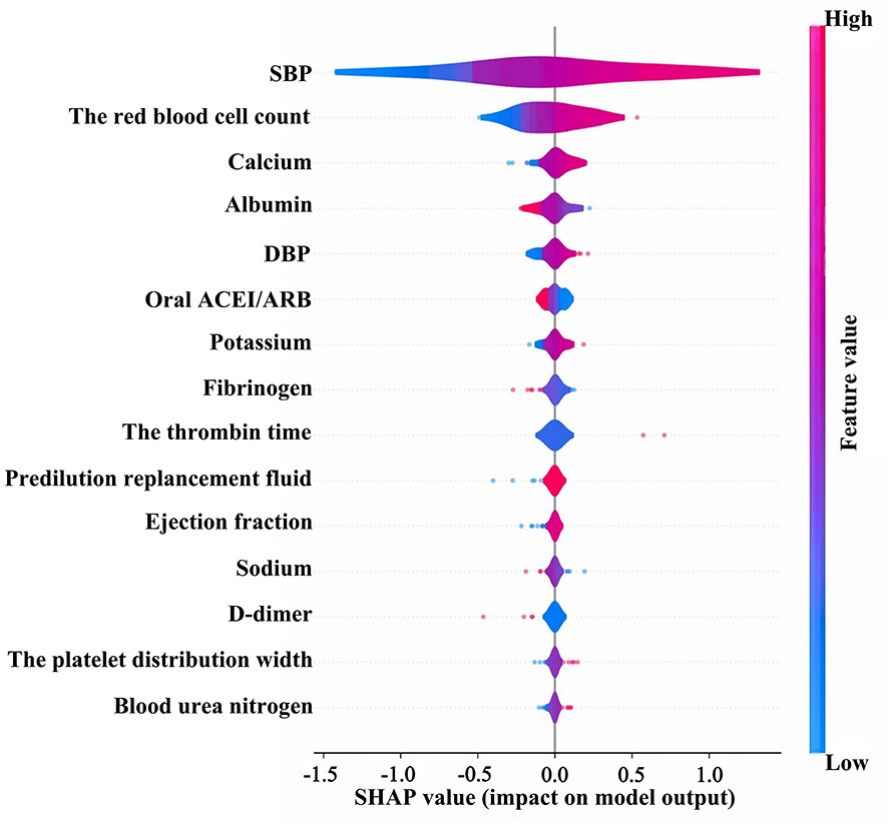
SHAP summary plot for the SVM model with the outcome defined as a reduction in SBP ≥20 mmHg from baseline. SVM, support vector machine; SBP, systolic blood pressure; DBP, diastolic blood pressure; ACEI/ARB, angiotensin-converting enzyme inhibitor/angiotensin II receptor blocker; SHAP, Shapley additive explanation.

**Figure 7:**
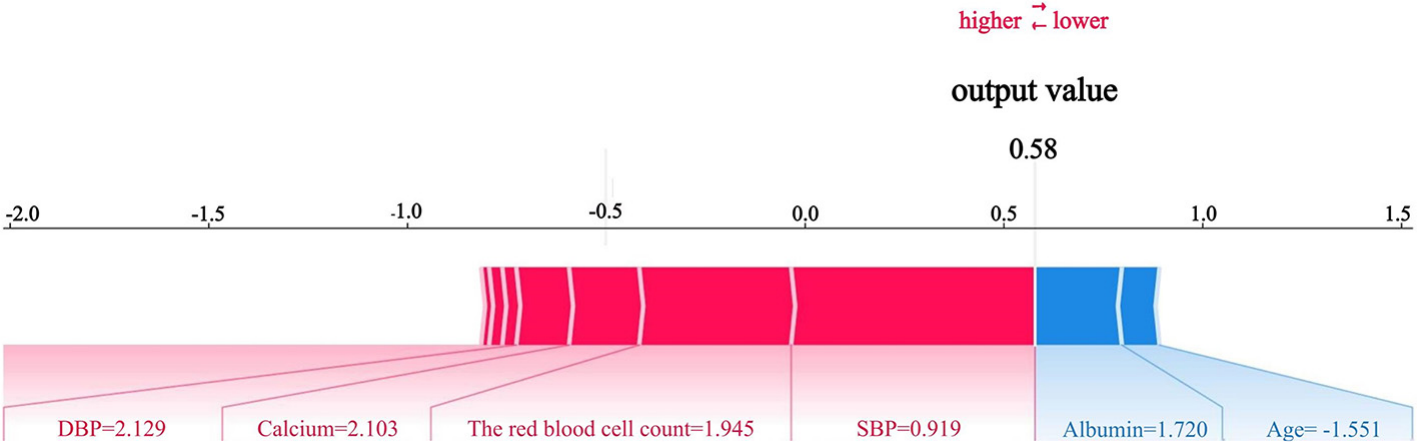
SHAP force plot for the SVM model with the outcome defined as a reduction in SBP ≥ 20 mmHg from baseline. SVM, support vector machine; SBP, systolic blood pressure; DBP, diastolic blood pressure; SHAP, Shapley additive explanation.

The SHAP method was applied to interpret the predictive models and to quantify the relative importance of individual features in predicting the occurrence of IDH after CRRT. As shown in Figures 6, 7, SBP was identified as the most influential predictor in the SVM model. Among laboratory parameters, red blood cell count was the greatest contributor, followed by albumin and calcium. In addition, variables such as DBP and the use of an oral ACEI/ARB also had a notable impact on the model predictions. To assess the stability of feature importance, we performed 500 bootstrap sampling analyses in the SVM model. The results showed that systolic blood pressure had the highest mean importance (0.6192) with a coefficient of variation of 0.2045, indicating high stability in its predictive contribution. Variables such as red blood cell count, DBP, albumin, and serum calcium ranked next in mean importance (range: 0.0592–0.1233), with coefficients of variation ranging from 0.5008 to 0.6988, details are shown in Supplementary Table 6. The overall ranking was highly consistent with the SHAP analysis results from the original study, further validating the robustness of the key predictors in our model.

## 4 Discussion

With the rapid advancement of artificial intelligence technologies, ML has been widely applied in prognostic prediction for patients with CKD (24–26). However, studies focusing on the prediction of IDH in patients with ESKD who are undergoing CRRT remain limited. To our knowledge, this study is the first to systematically evaluate the predictive performance of multiple ML algorithms in this specific clinical setting. The results demonstrated that the SVM model consistently outperformed the other five ML algorithms in our specific study cohort and validation design. Furthermore, model interpretation using SHAP provided clinically meaningful insights that may assist in decision-making. By identifying high-risk patients prior to CRRT initiation, clinicians can implement individualized early interventions targeting key contributing factors revealed by the model, such as baseline blood pressure and anemia status, thereby potentially reducing the incidence of IDH.

Currently, several studies have focused on predicting the occurrence of IDH. In a large-scale study conducted by Hanbi Lee et al. (27), a total of 943,220 haemodialysis sessions were analyzed, with an IDH incidence of 5.39%. Deep learning models demonstrated superior performance, and the most influential predictors included SBP, ultrafiltration quantity, and prior history of IDH. In a study by Min Woo Kang et al. (6) of 2,349 patients with AKI who received CRRT, pH, MAP, and SBP were identified as key predictors of IDH, with the XGBoost model achieving the best overall performance. Across different outcome definitions, the reported incidence of IDH ranged from 4% to 29%. Collectively, these studies support the effectiveness of ML approaches in predicting IDH and highlight their potential for clinical translation. However, existing studies have focused primarily on two populations: AKI patients receiving CRRT in the intensive care unit and patients with ESKD receiving conventional haemodialysis. Substantial differences exist between ESKD and AKI populations, as well as between CRRT and intermittent haemodialysis, in terms of patient characteristics and treatment mechanisms. First, the pathophysiological mechanisms underlying IDH differ fundamentally between ESKD and AKI patients. In ESKD, chronic volume overload, cardiac dysfunction, and autonomic nervous system impairment contribute to impaired vascular tone regulation and reduced cardiac output, predisposing patients to IDH during dialysis (28). In contrast, AKI patients often experience haemodynamic instability due to acute conditions such as infection, shock, or drug toxicity, leading to increased vascular permeability and more rapid intradialytic haemodynamic fluctuations (29). Second, differences in dialysis modality further influence the characteristics of IDH. CRRT, as a slow and continuous modality, facilitates more stable volume control and solute removal, whereas intermittent haemodialysis involves rapid ultrafiltration over a short period, which can precipitate abrupt intravascular volume depletion and severe IDH. Therefore, building upon prior research, the present study focused specifically on ESKD patients who underwent CRRT and applied multiple ML algorithms to develop predictive models for IDH risk in this distinct clinical population.

At present, there is no universally accepted diagnostic criterion for IDH. Previous studies have adopted heterogeneous definitions, primarily based on the nadir SBP, the absolute decline in SBP, the magnitude of MAP reduction, and/or the presence of accompanying symptoms (3, 16). Relying on a single criterion may therefore overlook clinically meaningful events. To enhance the robustness and clinical applicability of the predictive models, this study incorporated three widely used and clinically relevant definitions of IDH: an absolute decrease in SBP ≥ 20 mmHg, an absolute decrease in SBP ≥ 30 mmHg, and a reduction in MAP ≥ 10 mmHg from baseline. These definitions encompass different severities of IDH, and their combined use was intended to enable a more comprehensive capture of IDH events, thereby improving model performance across diverse clinical assessment scenarios. Clinical symptoms such as dizziness, fatigue, and nausea were not included in the definition of IDH, as these subjective reports are highly variable among individuals and may introduce bias. To ensure the objectivity and reproducibility of the outcomes, IDH was ultimately determined on the basis of objective haemodynamic parameters. The observation window was defined as the first 6 h after CRRT initiation for several reasons. First, prior studies have reported that the typical duration of a single CRRT session for patients with ESKD is approximately 6–8 h (3, 30, 31), and clinical practice at our institution predominantly adopts a 6-h treatment duration, making this time frame representative. More importantly, previous evidence indicates that the early phase of CRRT is characterized by the most pronounced degree of haemodynamic instability and the highest risk of IDH (6, 8, 27). Thus, selecting the first 6 h as the observation period allowed for the effective capture of critical risk while remaining clinically feasible. Across all three outcome definitions, the SVM model consistently demonstrated the best predictive performance, with AUC values ranging from 0.748 to 0.805, and showed favorable results in both the decision curve analysis and the calibration plots. Notably, even after further variable reduction using LASSO regression, the simplified SVM model maintained excellent performance. Overall, these findings indicate that the SVM model was the optimal predictive model among those tested in this study and was capable of effectively identifying patients at high risk of IDH prior to initiating CRRT. This enables clinicians to implement early, targeted interventions and optimize treatment strategies, potentially reducing the incidence of IDH. Our results are consistent with findings reported by Zhang et al. (32) and Zhuang et al. (33), who demonstrated the advantages of SVM-based models in nephrology research involving relatively small sample sizes and complex datasets. The superior performance of the SVM model may suggest that IDH during CRRT in ESKD patients is not driven by linear changes in a single factor but rather by complex, non-linear interactions between chronic comorbidities (such as cardiac dysfunction and autonomic neuropathy) and acute insults (such as infection and metabolic disturbances), a type of relationship that SVM is particularly well suited to model (34, 35).

To assess whether the added complexity of ML translated into clinical benefit, the SVM model was compared with LR. Under all three IDH definitions, the SVM achieved higher AUCs than the LR did, with significant differences confirmed by the DeLong test (all *P* < 0.05). DCA revealed that the SVM provided greater net benefit across most threshold probabilities, and the calibration curves with Brier scores indicated better predictive consistency. SHAP analysis further enhanced the interpretability of the SVM. Although the LR is structurally simpler, the SVM demonstrated superior predictive performance, clinical net benefit, and interpretability, suggesting that its added complexity offers meaningful clinical decision support value.

Further interpretation of the “black-box” model using SHAP revealed that SBP and DBP were among the most important variables associated with the occurrence of IDH, with SBP exerting a particularly strong influence. These findings are consistent with those of previous studies by Hanbi Lee et al. (27) and Min Woo Kang et al. (6). A possible explanation is that patients with ESKD commonly exhibit advanced vascular stiffening and impaired baroreflex sensitivity. Such a “rigid” circulatory system has a limited compensatory capacity in response to volume removal during CRRT, which predisposes patients to abrupt decreases in blood pressure and an increased risk of IDH (36). Moreover, SBP and DBP jointly reflect integrated physiological states, including cardiac pump function, systemic vascular resistance, and intravascular volume status (37). These findings underscore the clinical importance of individualized ultrafiltration target setting and enhanced haemodynamic monitoring in this patient population. Although baseline SBP is incorporated into the definition of IDH, its role as a core predictor is pathophysiologically justified and does not constitute model bias. IDH is diagnosed based on a relative reduction in blood pressure, which is inherently associated with baseline SBP. Meanwhile, the model relies on multiple indicators rather than SBP alone for prediction. The contribution of red blood cell count to IDH risk appeared relatively neutral, suggesting that its relationship with IDH is not simply linear and may be modulated by factors such as volume status, medication use, and underlying comorbidities. Notably, the study population consisted of ESKD patients, who are typically treated with erythropoietin and iron supplementation to correct anemia (3). Such widespread therapeutic interventions may maintain RBC counts within a relatively stable range, thereby attenuating its role as an independent risk factor for IDH. Hypoalbuminaemia was associated with an increased risk of IDH. Low serum albumin levels are often indicative of malnutrition, chronic inflammatory states, or hepatic dysfunction (38), all of which may lead to reduced effective circulation volume and compromised haemodynamic stability (39). These findings suggest that serum albumin may serve as a valuable predictor of IDH risk, highlighting the importance of pre-CRRT assessments of nutritional status and inflammation, as well as the consideration of colloid supplementation when appropriate. Although elevated calcium is expected to support blood pressure, we observed a positive association between pre-CRRT calcium levels and IDH risk, possibly due to dialysis-induced calcium fluctuations and uraemic endothelial dysfunction (40). This apparent inconsistency may be attributable to dialysis-induced calcium fluctuations, uraemia-related endothelial dysfunction, impaired autonomic regulation, and complex interactions with parathyroid hormone activity (41, 42). Collectively, these factors may blunt or even reverse the expected pressor effects of calcium, ultimately contributing to haemodynamic instability. In addition, several coagulation-related parameters, including fibrinogen level, prothrombin time, D-dimer level, thrombin time, and prothrombin activity, were also identified as important contributors to IDH risk. Abnormalities in these indices are frequently associated with conditions such as sepsis, multiple organ dysfunction, and hypercatabolic states (43), which are characterized by profound haemodynamic instability and an increased susceptibility to adverse IDH events. Clinically, heightened vigilance is warranted in such patients, and prompt identification and management of the underlying primary disease should be prioritized.

In this study, we employed multiple ML algorithms in combination with LASSO regression and SHAP to develop a predictive model for IDH in patients with ESKD initiating CRRT, aiming to provide a data-driven tool to support clinical decision-making. Nevertheless, several limitations should be acknowledged. First, the single-center design may limit the external validity and generalizability of the model. Although temporal validation assessed the model's robustness against temporal drift, it cannot substitute for geographic or institutional external validation, and the single-center design may still lead to overestimation of model performance. Future multicenter, large-scale cohort studies are warranted to further validate and enhance the robustness and applicability of the model. Second, the current model has not yet been integrated into a clinical information system and has not undergone prospective validation in real-world clinical settings. Future work may focus on embedding the model into clinical informatics platforms to enable the real-time extraction of baseline characteristics, laboratory data, and treatment parameters, thereby facilitating automated risk stratification and early warning. Third, in this study, only the first CRRT session for each patient was included to avoid duplicate data. Additionally, due to the limitations of data completeness in this retrospective design, a history of previous IDH was not incorporated into the prediction model. Furthermore, patients who were on vasoactive agents at CRRT initiation or required short-acting antihypertensive medications during treatment were excluded. Although this exclusion strategy helped minimize confounding effects from established hemodynamic interventions and facilitated the identification of other potential risk factors, it also restricted the generalizability of the model to some extent. Future studies may employ stratified analyses or develop specific submodels for these high-risk populations, standardize the collection of key information such as prior IDH history, and conduct external validation to extend the model to a broader patient spectrum, thereby further improving its predictive performance. Fourth, this study focused primarily on the risk of IDH and did not incorporate other CRRT-related outcomes, such as mortality. Future studies should expand the predictive scope to construct comprehensive risk assessment frameworks that integrate multiple clinically relevant outcomes. Fifth, our model was developed based solely on static baseline characteristics prior to CRRT initiation, without incorporating dynamic intra-treatment blood pressure changes or prior history of IDH. Future studies should integrate dynamic data to further refine the predictive model. In addition, although class weighting and AUPRC evaluation were used to reduce the interference of class imbalance, residual bias caused by uneven sample distribution could not be entirely avoided. Ongoing attention to advances in this field and the exploration of more sophisticated and efficient ML techniques are needed to further improve model performance (44), ultimately advancing intelligent, personalized, and comprehensive clinical decision support.

## 5 Conclusion

This study demonstrates that, within the examined cohort of ESKD patients following CRRT initiation, the SVM model performed robustly in predicting the risk of IDH. In addition, SBP and red blood cell count were identified as key factors associated with the occurrence of IDH. DCA and SHAP further provided strong support for clinical decision-making by enhancing model interpretability and clinical utility. These findings highlight the substantial potential of machine learning approaches in predicting complications related to CRRT in ESKD patients and in optimizing individualized treatment strategies.

## Data Availability

The raw data supporting the conclusions of this article will be made available by the authors, without undue reservation.
