# Supplementary material for: Machine learning model for predicting hypotension following continuous renal replacement therapy initiation in end-stage kidney disease patients: a SHAP-interpretable approach
Source: Front Med (Lausanne). 2026 May 15;13:1807513. doi: 10.3389/fmed.2026.1807513 (PMC13219051; doi:10.3389/fmed.2026.1807513)
Supplement: Supplementary file 1 [file Table_1.doc]

**Supplementary Table S1** The hyperparameter tuning procedure of the ML methods

| **Model** | **Candidate hyperparameter ranges** | **Search strategy** | **Optimal hyperparameter combination** |
| --- | --- | --- | --- |
| SVM | Regularization coefficient C: [0.01, 0.1, 1, 10, 100] | Grid search | 1 |
|  | Kernel coefficient gamma: [scale, 0.001, 0.01, 0.1, 1] |  | scale |
|  | Kernel function: [rbf, linear] |  | rbf |
| DT | Maximum depth: [3, 5, 7, 10, 15, None] | Grid search | 5 |
|  | Minimum samples split: [2, 5, 10, 20] |  | 10 |
|  | Minimum samples leaf: [1, 2, 5, 10] |  | 2 |
| RF | Number of trees: [50, 100, 200, 300] | Grid search | 200 |
|  | Maximum depth: [5, 10, 15, 20, None] |  | 5 |
|  | Minimum samples split: [2, 5, 10] |  | 5 |
|  | Minimum samples leaf: [1, 2, 4] |  | 2 |
|  | Maximum features: [sqrt, log2, None] |  | None |
| GBM | Number of weak learners: [50, 100, 200, 300] | Grid search | 200 |
|  | Learning rate: [0.001, 0.01, 0.1, 0.2, 0.3] |  | 0.01 |
|  | Maximum depth: [3, 5, 7] |  | 3 |
|  | Minimum samples split: [2, 5, 10] |  | 5 |
| XGBoost | Number of trees: [50, 100, 200, 300] | Grid search | 200 |
|  | Maximum depth: [3, 5, 7, 9] |  | 5 |
|  | Learning rate: [0.001, 0.01, 0.1, 0.2, 0.3] |  | 0.1 |
| LR | Regularization coefficient C: [0.001, 0.01, 0.1, 1, 10, 100] | Grid search | 0.1 |
|  | Regularization type: [L1, L2] |  | L1 |

**Abbreviations:** SVM: support vector machine; DT: decision tree; RF: random forest; GBM: gradient boosting machine; XGBoost: extreme gradient boosting; LR: logistic regression

**Supplementary Table S2** Comparison of variables before and after multiple imputation

| **Characteristic** | **Missing value ratio (%)** | **Before imputation** | **After imputation** | **P value** | **Dataset** |
| --- | --- | --- | --- | --- | --- |
| Weight (kg) | 0.518 | 66.363±14.149 | 66.37±14.113 | 0.989 | Training |
| Lymphocyte ratio (%) | 0.130 | 13.835±7.573 | 13.834±7.568 | 0.997 | Training |
| Red blood cell count (×10^12^/L) | 0.130 | 3.179±0.773 | 3.179±0.772 | 0.998 | Training |
| Platelet count (×10^9^/L) | 0.130 | 181.486±83.074 | 181.466±83.022 | 0.996 | Training |
| Platelet distribution width | 0.130 | 16.218±0.462 | 16.218±0.462 | 0.998 | Training |
| Direct bilirubin (umol/L) | 1.684 | 4.987±25.425 | 4.976±25.214 | 0.994 | Training |
| Indirect bilirubin (umol/L) | 1.684 | 4.171±4.168 | 4.166±4.133 | 0.979 | Training |
| Aspartate aminotransferase (U/L) | 4.145 | 34.817±92.789 | 34.570±90.926 | 0.958 | Training |
| Alanine aminotransferase (U/L) | 1.295 | 37.035±125.223 | 36.890±124.417 | 0.982 | Training |
| Alkaline phosphatase (U/L) | 4.534 | 103.582±73.088 | 103.138±71.471 | 0.905 | Training |
| Albumin (g/L) | 2.332 | 34.420±6.106 | 34.423±6.036 | 0.993 | Training |
| Blood urea nitrogen (mmol/L) | 1.554 | 24.256±10.233 | 24.248±10.155 | 0.989 | Training |
| Creatinine (umol/L) | 1.036 | 834.443±412.134 | 834.235±410.047 | 0.992 | Training |
| Uric acid (umol/L) | 14.637 | 427.105±206.701 | 425.383±191.407 | 0.871 | Training |
| Glucose (mmol/L) | 8.290 | 8.456±5.432 | 8.402±5.209 | 0.845 | Training |
| Calcium (mmol/L) | 0.648 | 2.134±0.266 | 2.134±0.265 | 0.999 | Training |
| Phosphate (mmol/L) | 1.036 | 1.907±0.775 | 1.907±0.771 | 0.994 | Training |
| Potassium (mmol/L) | 0.130 | 4.724±0.922 | 4.724±0.921 | 0.999 | Training |
| Sodium (mmol/L) | 0.130 | 137.308±4.509 | 137.307±4.506 | 0.999 | Training |
| Chloride (mmol/L) | 0.259 | 99.724±5.528 | 99.724±5.528 | 0.991 | Training |
| Carbon dioxide (mmol/L) | 0.259 | 21.790±5.384 | 21.792±5.377 | 0.994 | Training |
| Cholesterol (mmol/L) | 9.067 | 3.925±1.414 | 3.9144±1.3506 | 0.884 | Training |
| Triglycerides (mmol/L) | 8.808 | 1.860±1.373 | 1.847±1.312 | 0.859 | Training |
| High-density lipoprotein (mmol/L) | 9.197 | 0.984±0.402 | 0.982±0.384 | 0.895 | Training |
| Low-density lipoprotein (mmol/L) | 8.938 | 2.069±0.916 | 2.062±0.875 | 0.889 | Training |
| Prothrombin time (s) | 1.295 | 13.148±2.834 | 13.145±2.816 | 0.985 | Training |
| p-Prothrombin time activity (%) | 1.943 | 105.261±104.210 | 105.179±103.223 | 0.988 | Training |
| Activated partial thromboplastin time (s) | 1.425 | 31.282±9.287 | 31.270±9.222 | 0.981 | Training |
| Fibrinogen (g/L) | 1.425 | 4.375±1.577 | 4.375±1.566 | 0.999 | Training |
| Thrombin time (s) | 1.684 | 18.655±12.761 | 18.659±12.658 | 0.995 | Training |
| D-dimer (mg/L) | 2.202 | 3.125±4.972 | 3.110±4.918 | 0.954 | Training |
| Intact parathyroid hormone (pg/mL) | 8.161 | 482.872±479.003 | 480.718±459.773 | 0.930 | Training |
| Ejection fraction | 10.233 | 0.562±0.105 | 0.563±0.100 | 0.845 | Training |
| Weight (kg) | 0.302 | 66.881±14.485 | 66.867±14.465 | 0.991 | Testing |
| Direct bilirubin (umol/L) | 2.719 | 3.883±10.475 | 3.847±10.333 | 0.965 | Testing |
| Indirect bilirubin (umol/L) | 2.719 | 4.421±5.233 | 4.401±5.163 | 0.960 | Testing |
| Aspartate aminotransferase (U/L) | 6.042 | 28.730±67.554 | 28.233±65.506 | 0.925 | Testing |
| Alanine aminotransferase (U/L) | 2.115 | 33.000±124.312 | 32.759±122.998 | 0.980 | Testing |
| Alkaline phosphatase (U/L) | 6.647 | 95.792±65.137 | 95.231±62.979 | 0.911 | Testing |
| Albumin (g/L) | 3.021 | 35.238±6.818 | 35.242±6.714 | 0.994 | Testing |
| Blood urea nitrogen (mmol/L) | 1.813 | 24.165±10.104 | 24.160±10.015 | 0.995 | Testing |
| Creatinine (umol/L) | 0.906 | 834.472±397.912 | 834.517±396.106 | 0.999 | Testing |
| Uric acid (umol/L) | 10.574 | 424.788±164.076 | 423.676±155.365 | 0.931 | Testing |
| Glucose (mmol/L) | 8.761 | 8.235±4.386 | 8.178±4.198 | 0.870 | Testing |
| Phosphate (mmol/L) | 0.604 | 1.872±0.771 | 1.871±0.769 | 0.990 | Testing |
| Cholesterol (mmol/L) | 8.761 | 3.940±1.472 | 3.927±1.407 | 0.909 | Testing |
| Triglycerides (mmol/L) | 8.761 | 1.826±1.230 | 1.817±1.177 | 0.929 | Testing |
| High-density lipoprotein (mmol/L) | 8.459 | 1.030±0.421 | 1.029±0.403 | 0.962 | Testing |
| Low-density lipoprotein (mmol/L) | 8.761 | 2.0470±0.859 | 2.039±0.822 | 0.909 | Testing |
| Prothrombin time (s) | 0.604 | 13.013±2.024 | 13.014±2.019 | 0.994 | Testing |
| p-Prothrombin time activity (%) | 0.906 | 103.672±100.593 | 103.718±100.149 | 0.995 | Testing |
| Activated partial thromboplastin time (s) | 0.604 | 31.162±7.307 | 31.153±7.286 | 0.988 | Testing |
| Fibrinogen (g/L) | 0.604 | 4.321±1.515 | 4.320±1.511 | 0.996 | Testing |
| Thrombin time (s) | 0.604 | 18.371±6.074 | 18.369±6.056 | 0.996 | Testing |
| D-dimer (mg/L) | 1.511 | 2.833±4.712 | 2.821±4.677 | 0.974 | Testing |
| Intact parathyroid hormone (pg/mL) | 9.970 | 501.795±459.014 | 496.667±436.299 | 0.886 | Testing |
| Ejection fraction | 9.063 | 0.557±0.108 | 0.559±0.103 | 0.871 | Testing |

**Supplementary Table S3** Diagnosis of predictor sparsity and multicollinearity

| **Characteristic** | **Variance** | **VIF** | **Reason for exclusion** |
| --- | --- | --- | --- |
| Female, n (%) | 0.210 | 1.598 |  |
| Age (years) | 171.292 | 1.867 |  |
| SBP (mmHg) | 679.944 | 113873.168 | High VIF |
| DBP (mmHg) | 237.453 | 145348.946 | High VIF |
| MAP (mmHg) | 284.053 | 405703.679 | High VIF |
| Weight (kg) | 202.948 | 1.770 |  |
| Vascular access, n (%) | 0.536 | 3.152 |  |
| Blood dialysis filter, n (%) | 0.481 | 1.778 |  |
| Heart rate (beats/minute) | 277.880 | 1.298 |  |
| Potassium in replacement fluid (mmol/L) | 11.703 | 2.071 |  |
| Sodium bicarbonate infusion rate during CRRT (ml/h) | 232.841 | 3.284 |  |
| Treatment model (CVVH), n (%) | 0.299 | 3.219 |  |
| Blood flow rate (ml/min) | 70.759 | 1.218 |  |
| Predilution replacement fluid (ml/h) | 8480.518 | 1.699 |  |
| Postdilution replacement fluid (ml/h) | 19024.489 | 2.858 |  |
| Ultrafiltration quantity (ml/h) | 22603.901 | 1.294 |  |
| White blood cell count (×10⁹/L) | 15.815 | 1.918 |  |
| Neutrophil ratio (%) | 118.955 | 3.590 |  |
| Lymphocyte ratio (%) | 59.437 | 3.632 |  |
| Red blood cell (×10^12^/L) | 0.626 | 9.763 |  |
| Haemoglobin (g/L) | 498.450 | 10.542 | High VIF |
| Haematocrit (L/L) | 0.005 |  | Near-zero variance |
| Platelet count (×10⁹/L) | 6896.413 | 1.750 |  |
| Platelet distribution width | 0.211 | 1.361 |  |
| Direct bilirubin (umol/L) | 486.566 | 2.005 |  |
| Indirect bilirubin (umol/L) | 20.344 | 2.139 |  |
| Aspartate aminotransferase (U/L) | 7414.673 | 1.727 |  |
| Alanine aminotransferase (U/L) | 15602.058 | 1.821 |  |
| Alkaline phosphatase (U/L) | 5025.401 | 1.346 |  |
| Albumin (g/L) | 40.128 | 1.965 |  |
| Blood urea nitrogen (mmol/L) | 103.840 | 3.012 |  |
| Creatinine (umol/L) | 166245.737 | 2.753 |  |
| Uric acid (umol/L) | 37794.508 | 1.728 |  |
| Glucose (mmol/L) | 26.424 | 1.540 |  |
| Calcium (mmol/L) | 0.073 | 2.059 |  |
| Phosphate (mmol/L) | 0.599 | 2.189 |  |
| Potassium (mmol/L) | 0.823 | 2.295 |  |
| Sodium (mmol/L) | 19.623 | 2.976 |  |
| Chloride (mmol/L) | 31.271 | 4.028 |  |
| Carbon dioxide (mmol/L) | 28.939 | 2.536 |  |
| Cholesterol (mmol/L) | 2.048 | 4.139 |  |
| Triglycerides (mmol/L) | 1.772 | 1.837 |  |
| High-density lipoprotein (mmol/L) | 0.167 | 1.862 |  |
| Low-density lipoprotein (mmol/L) | 0.808 | 3.397 |  |
| Prothrombin time (s) | 6.844 | 14.809 | High VIF |
| International normalized ratio | 0.060 | 13.569 | High VIF |
| p-Prothrombin time activity (%) | 10,626.724 | 1.117 |  |
| Activated partial thromboplastin time (s) | 76.270 | 2.021 |  |
| Fibrinogen (g/L) | 2.427 | 1.680 |  |
| Thrombin time (s) | 124.705 | 1.210 |  |
| D-dimer (mg/L) | 23.956 | 1.387 |  |
| Intact parathyroid hormone (pg/mL) | 223,755.220 | 1.360 |  |
| Ejection fraction | 0.011 | 1.397 |  |
| Dialysis time (month) | 3269.278 | 1.659 |  |
| Anticoagulant use, n (%) | 0.191 | 1.228 |  |
| Hypertension, n (%) | 0.070 | 1.254 |  |
| Diabetes mellitus, n (%) | 0.250 | 1.659 |  |
| Coronary heart disease, n (%) | 0.237 | 1.343 |  |
| Arrhythmia, n (%) | 0.101 | 1.242 |  |
| Stroke, n (%) | 0.166 | 1.168 |  |
| Oral CCB, n (%) | 0.247 | 1.487 |  |
| Oral ACEI/ARB, n (%) | 0.250 | 1.364 |  |
| Oral β-blocker, n (%) | 0.250 | 1.257 |  |

**Abbreviations:** SBP: systolic blood pressure; DBP: diastolic blood pressure; MAP (mean arterial pressure); CRRT: continuous renal replacement therapy; CVVH: continuous venovenous haemofiltration; CCB: calcium channel blocker; ACEI/ARB: angiotensin-converting enzyme inhibitor/angiotensin II receptor blocker

**Supplementary Table S4** Difference in AUC compared to the reference model (SVM) across different outcome definitions

| **Model** | **SBP ↓ ≥20 mmHg** | | | **SBP ↓ ≥30 mmHg** | | | **MAP ↓ ≥10 mmHg** | | |
| --- | --- | --- | --- | --- | --- | --- | --- | --- | --- |
|  | **ΔAUC** | **95%CI** | **P value** | **ΔAUC** | **95%CI** | **P value** | **ΔAUC** | **95%CI** | **P value** |
| **SVM (Reference)** | - | - | - | - | - | - | - | - | - |
| **XGBoost** | 0.060 | 0.023-0.097 | 0.002 | 0.017 | 0.003-0.064 | 0.048 | 0.061 | 0.034-0.087 | 0.001 |
| **GBM** | 0.032 | 0.016-0.080 | 0.019 | 0.031 | 0.015-0.076 | 0.019 | 0.038 | 0.012-0.063 | 0.004 |
| **RF** | 0.044 | 0.011-0.077 | 0.010 | 0.029 | 0.017-0.073 | 0.022 | 0.037 | 0.014-0.058 | 0.001 |
| **DT** | 0.051 | 0.001-0.101 | 0.044 | 0.077 | 0.018-0.134 | 0.010 | 0.080 | 0.047-0.112 | 0.001 |
| **LR** | 0.017 | 0.011-0.045 | 0.041 | 0.008 | 0.002-0.036 | 0.046 | 0.012 | 0.006-0.028 | 0.022 |

ΔAUC represents the difference in AUC between the SVM (reference) and each of the other models. **Abbreviations:** SVM: support vector machine; SBP: systolic blood pressure; MAP: mean arterial pressure; XGBoost: extreme gradient boosting; GBM: gradient boosting machine; RF: random forest; DT: decision tree; LR: logistic regression; AUC: area under the curve

**Supplementary Table S5** Model performance metrics by time-ordered data split (IDH defined as SBP reduction ≥20 mmHg from baseline)

| **Model** | **AUC (95%CI)** | **Sensitivity (95%CI)** | **Specificity (95%CI)** | **PPV (95%CI)** | **NPV (95%CI)** | **F1 score (95%CI)** | **Brier's score (95%CI)** |
| --- | --- | --- | --- | --- | --- | --- | --- |
| SVM | 0.778 (0.724-0.830) | 0.782 (0.720-0.844) | 0.860 (0.811-0.903) | 0.741 (0.667-0.811) | 0.887 (0.847-0.922) | 0.761 (0.708-0.809) | 0.178 (0.159-0.197) |
| XGBoost | 0.722 (0.660-0.781) | 0.745 (0.687-0.803) | 0.850 (0.802-0.897) | 0.717 (0.636-0.789) | 0.867 (0.825-0.906) | 0.731 (0.672-0.786) | 0.191 (0.171-0.211) |
| GBM | 0.736 (0.678-0.792) | 0.800 (0.740-0.860) | 0.874 (0.828-0.915) | 0.771 (0.699-0.837) | 0.892 (0.854-0.927) | 0.785 (0.729-0.836) | 0.191 (0.169-0.213) |
| RF | 0.732 (0.670-0.786) | 0.700 (0.642-0.758) | 0.813 (0.756-0.864) | 0.677 (0.597-0.752) | 0.827 (0.781-0.870) | 0.688 (0.627-0.748) | 0.190 (0.171-0.210) |
| DT | 0.714 (0.651-0.772) | 0.782 (0.733-0.831) | 0.807 (0.766-0.844) | 0.816 (0.750-0.873) | 0.877 (0.839-0.913) | 0.799 (0.752-0.842) | 0.198 (0.183-0.214) |
| LR | 0.754 (0.695-0.808) | 0.800 (0.751-0.849) | 0.874 (0.825-0.916) | 0.768 (0.702-0.828) | 0.893 (0.856-0.927) | 0.784 (0.735-0.829) | 0.184 (0.164-0.204) |

**Abbreviations:** IDH: intradialytic hypotension; SBP: systolic blood pressure; SVM: support vector machine; XGBoost: extreme gradient boosting; GBM: gradient boosting machine; RF: random forest; DT: decision tree; LR: logistic regression; AUC: area under the curve; PPV: positive predictive value; NPV: negative predictive value

Supplementary Table S6 Bootstrap-Based Stability Analysis for Feature Importance (IDH defined as SBP reduction ≥20 mmHg from baseline)

| **Rank** | **Feature** | **Mean Importance** | **Std Importance** | **95% CI Lower** | **95% CI Upper** | **CV** |
| --- | --- | --- | --- | --- | --- | --- |
| 1 | SBP | 0.619 | 0.127 | 0.421 | 0.895 | 0.205 |
| 2 | Red blood cell count | 0.123 | 0.062 | 0.012 | 0.253 | 0.501 |
| 3 | DBP | 0.061 | 0.042 | 0.005 | 0.145 | 0.695 |
| 4 | Albumin | 0.060 | 0.042 | 0.003 | 0.160 | 0.699 |
| 5 | Calcium | 0.059 | 0.041 | 0.004 | 0.142 | 0.695 |
| 6 | Triglycerides | 0.046 | 0.037 | 0.002 | 0.124 | 0.814 |
| 7 | Prothrombin time | 0.042 | 0.031 | 0.001 | 0.107 | 0.768 |
| 8 | Fibrinogen | 0.041 | 0.031 | 0.001 | 0.107 | 0.768 |
| 9 | Potassium | 0.040 | 0.034 | 0.002 | 0.131 | 0.853 |
| 10 | Platelet distribution width | 0.038 | 0.035 | 0.001 | 0.120 | 0.937 |
| 11 | Ultrafiltration quantity | 0.033 | 0.034 | 0.001 | 0.123 | 1.015 |
| 12 | Ejection fraction | 0.032 | 0.028 | 0.001 | 0.101 | 0.859 |
| 13 | Age | 0.031 | 0.027 | 0.002 | 0.105 | 0.894 |
| 14 | Oral ACEI/ARB | 0.029 | 0.026 | 0.001 | 0.085 | 0.903 |
| 15 | Dialysis time | 0.026 | 0.026 | 0.001 | 0.095 | 1.007 |

A bootstrap resampling procedure with 500 iterations was performed to evaluate the stability of feature importance. Abbreviations: IDH: intradialytic hypotension; SBP: systolic blood pressure; DBP: diastolic blood pressure; ACEI/ARB: angiotensin-converting enzyme inhibitor/angiotensin II receptor blocker; CV: coefficient of variation

**Supplementary Table S7** Model performance metrics after LASSO feature selection (IDH defined as a reduction in SBP ≥20 mmHg from baseline)

| **Model** | **AUC** | **Sensitivity** | **Specificity** | **PPV** | **NPV** | **F1 score** | **Brier's score** |
| --- | --- | --- | --- | --- | --- | --- | --- |
| SVM | 0.762 | 0.804 | 0.839 | 0.720 | 0.766 | 0.760 | 0.182 |
| XGBoost | 0.697 | 0.754 | 0.876 | 0.697 | 0.723 | 0.724 | 0.198 |
| GBM | 0.738 | 0.787 | 0.839 | 0.711 | 0.752 | 0.747 | 0.192 |
| RF | 0.733 | 0.760 | 0.849 | 0.712 | 0.752 | 0.735 | 0.189 |
| DT | 0.713 | 0.719 | 0.917 | 0.767 | 0.722 | 0.742 | 0.199 |
| LR | 0.756 | 0.813 | 0.835 | 0.717 | 0.768 | 0.762 | 0.184 |

**Abbreviations:** LASSO: least absolute shrinkage and selection operator; IDH: intradialytic hypotension; SBP: systolic blood pressure; SVM: support vector machine; XGBoost: extreme gradient boosting; GBM: gradient boosting machine; RF: random forest; DT: decision tree; LR: logistic regression; AUC: area under the curve; PPV: positive predictive value; NPV: negative predictive value

**Supplementary Table S8** Model performance metrics after LASSO feature selection (IDH defined as a reduction in SBP ≥30 mmHg from baseline)

| **Model** | **AUC** | **Sensitivity** | **Specificity** | **PPV** | **NPV** | **F1 score** | **Brier's score** |
| --- | --- | --- | --- | --- | --- | --- | --- |
| SVM | 0.809 | 0.859 | 0.874 | 0.767 | 0.839 | 0.810 | 0.120 |
| XGBoost | 0.765 | 0.850 | 0.863 | 0.615 | 0.843 | 0.714 | 0.128 |
| GBM | 0.801 | 0.844 | 0.863 | 0.688 | 0.860 | 0.758 | 0.122 |
| RF | 0.790 | 0.819 | 0.878 | 0.700 | 0.839 | 0.755 | 0.125 |
| DT | 0.767 | 0.850 | 0.878 | 0.727 | 0.845 | 0.784 | 0.127 |
| LR | 0.774 | 0.850 | 0.866 | 0.740 | 0.843 | 0.791 | 0.126 |

**Abbreviations:** LASSO: least absolute shrinkage and selection operator; IDH: intradialytic hypotension; SBP: systolic blood pressure; SVM: support vector machine; XGBoost: extreme gradient boosting; GBM: gradient boosting machine; RF: random forest; DT: decision tree; LR: logistic regression; AUC: area under the curve; PPV: positive predictive value; NPV: negative predictive value

**Supplementary Table S9** Model performance metrics after LASSO feature selection (IDH defined as a reduction in MAP ≥10 mmHg from baseline)

| **Model** | **AUC** | **Sensitivity** | **Specificity** | **PPV** | **NPV** | **F1 score** | **Brier's score** |
| --- | --- | --- | --- | --- | --- | --- | --- |
| SVM | 0.744 | 0.709 | 0.784 | 0.762 | 0.759 | 0.734 | 0.206 |
| XGBoost | 0.690 | 0.627 | 0.735 | 0.738 | 0.714 | 0.678 | 0.218 |
| GBM | 0.722 | 0.679 | 0.709 | 0.742 | 0.736 | 0.709 | 0.213 |
| RF | 0.726 | 0.664 | 0.770 | 0.778 | 0.746 | 0.716 | 0.211 |
| DT | 0.676 | 0.649 | 0.719 | 0.737 | 0.722 | 0.691 | 0.226 |
| LR | 0.734 | 0.687 | 0.770 | 0.786 | 0.759 | 0.733 | 0.211 |

**Abbreviations:** LASSO: least absolute shrinkage and selection operator; IDH: intradialytic hypotension; MAP: mean arterial pressure; SVM: support vector machine; XGBoost: extreme gradient boosting; GBM: gradient boosting machine; RF: random forest; DT: decision tree; LR: logistic regression; AUC: area under the curve; PPV: positive predictive value; NPV: negative predictive value
