# Supplementary figures and images for: Machine learning model for predicting hypotension following continuous renal replacement therapy initiation in end-stage kidney disease patients: a SHAP-interpretable approach
Source: Front Med (Lausanne). 2026 May 15;13:1807513. doi: 10.3389/fmed.2026.1807513 (PMC13219051; doi:10.3389/fmed.2026.1807513)

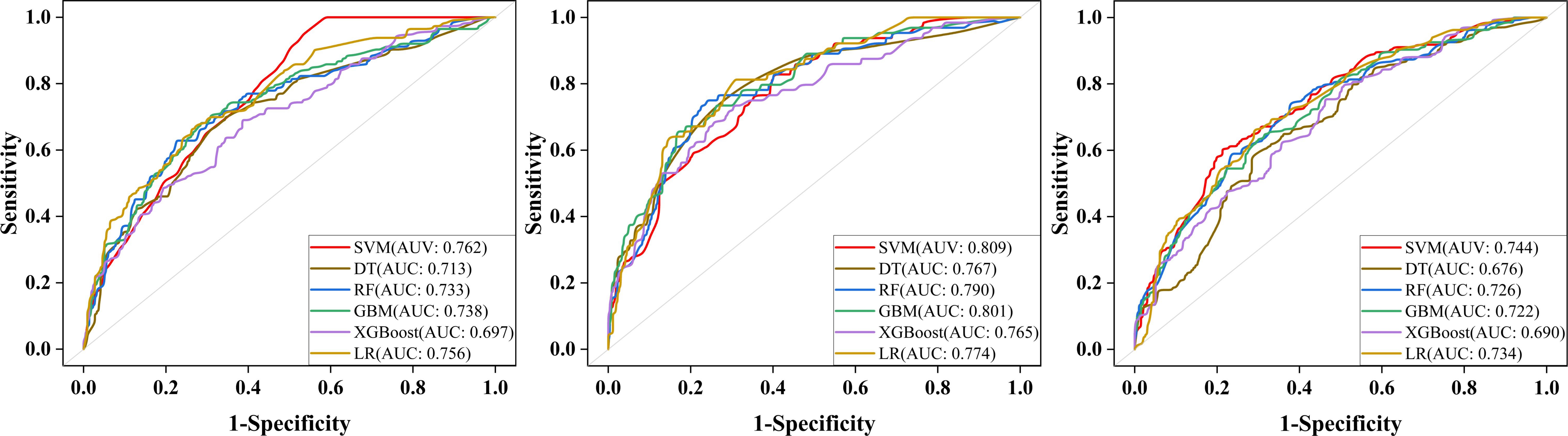

Supplement: Supplementary file 6 [file Image_5.jpeg]
